# Sulfur hexafluoride: MAK Value Documentation, addendum – Translation of the German version from 2023

**DOI:** 10.34865/mb255162e10_1ad

**Published:** 2025-03-31

**Authors:** Andrea Hartwig, MAK Commission

**Affiliations:** 1 Institute of Applied Biosciences. Department of Food Chemistry and Toxicology. Karlsruhe Institute of Technology (KIT) Adenauerring 20a, Building 50.41 76131 Karlsruhe Germany; 2 Permanent Senate Commission for the Investigation of Health Hazards of Chemical Compounds in the Work Area. Deutsche Forschungsgemeinschaft, Kennedyallee 40, 53175 Bonn, Germany. Further information: Permanent Senate Commission for the Investigation of Health Hazards of Chemical Compounds in the Work Area | DFG

**Keywords:** sulfur hexafluoride, inert gas, inhalation, MAK value, maximum workplace concentration

## Abstract

The German Commission for the Investigation of Health Hazards of Chemical Compounds in the Work Area (MAK Commission) has re-evaluated the occupational exposure limit value (maximum concentration at the workplace, MAK value) of sulfur hexafluoride [2551-62-4] considering all toxicological end points. Relevant studies were identified from a literature search and also unpublished study reports were used. This addendum evaluates only pure sulfur hexafluoride. Reaction products that are highly toxic for humans can form during its use as an insulating gas in high voltage switchgear. Under normal conditions, the reactivity of sulfur hexafluoride gas is comparable to that of helium or nitrogen. Therefore, systemic or local effects were not observed in studies carried out in compliance with OECD test guidelines with exposure of rats to the gas at concentrations up to 20 052 ml/m^3^ for 13 weeks. Acute exposure to 550 000 ml/m^3^ (55%) induced sleepiness and analgesia in humans. On the basis of the NOAEC of 20 052 ml/m^3^, a maximum concentration at the workplace (MAK value) of 5000 ml/m^3^ has been set. As significant effects were not observed in humans up to 390 000 ml/m^3^, Peak Limitation Category II and the excursion factor of 8 have been retained. Sulfur hexafluoride showed no genotoxic potential. Carcinogenicity studies have not been carried out. Sulfur hexafluoride was not teratogenic in a developmental toxicity study in concentrations up to 19 100 ml/m^3^, but led to a transient delay in foetal development. After consideration of all data as well as the fact that sulfur hexafluoride is inert under normal conditions, it has been assigned to Pregnancy Risk Group C. There are no data for the sensitizing potential of sulfur hexafluoride. Skin contact is not expected to contribute significantly to systemic toxicity.

**Table:** 

**MAK value (2022)**	**5000 ml/m^3^ (ppm) ≙ 30 000 mg/m^3^**
**Peak limitation (2002)**	**Category II, excursion factor 8**

**Absorption through the skin**	**–**
**Sensitization**	**–**
**Carcinogenicity**	**–**
**Prenatal toxicity (2022)**	**Pregnancy Risk Group C**
**Germ cell mutagenicity**	**–**

**BAT value**	**–**

Synonyms	sulfur fluoride sulfur(VI) fluoride sulphur fluoride
Chemical name (IUPAC)	hexafluoro-λ^6^-sulfane
CAS number	2551-62-4
Molecular formula	SF_6_
Molar mass	146.05 g/mol
Sublimation temperature at 1013 hPa	–63.8 °C (ECHA 2020)
Melting point at 1013 hPa	–50.8 °C (ECHA 2020)
Vapour pressure at 25 °C	23 700 hPa (ECHA 2020)
log K_OW_ at 20 °C	1.68 (ECHA 2020)
Solubility at 25 °C	31 mg/l water (ECHA 2020)
Hydrolytic stability	no data
Production	from elemental sulfur in a stream of fluorine gas, any further sulfur fluorides that form are disproportionated at high temperature (400 °C) and by washing the gas in alkaline solution (SF_6_ is inert in this solution, washing removes other compounds), pure sulfur hexafluoride is then extracted by pressure distillation (NCBI 2022)
Uses	in the electrical industry, equipment manufacturing, as an etching gas in the semiconductor manufacturing industry, for the manufacturing of optical glass fibres, in aluminium and magnesium foundries, research institutes (Statistisches Bundesamt 2020), insulating gas in medium-voltage and high-voltage technology (Dervos and Vassiliou 2000), contrast agent in ultrasound imaging (EMA 2006), gas tamponade (about 20%) during eye surgery (Amara et al. 2021; Tan et al. 2013), tracer gas in wind channels (NCBI 2022)
**1 ml/m^3^ (ppm) ≙ 6.06 mg/m^3^**	**1 mg/m^3^ ≙ 0.165 ml/m^3^ (ppm)**

Note: The evaluation refers to the pure substance; highly toxic decomposition and reaction products may develop from sulfur hexafluoride at a very high energy input (for example, electrical discharges or at temperatures higher than 500 °C).

Documentation for sulfur hexafluoride was published in 1972 (Henschler 1972, available in German only). Cited unpublished toxicological studies from companies have been made available to the Commission.

Sulfur hexafluoride is an odourless gas that is chemically inert. It is used mainly as an insulating gas in medium- and high-voltage switches because of its high dielectric strength and its good arc-quenching capability. Furthermore, it is used as an etching gas in the manufacture of semiconductor components and for cleaning applications involving etching (ECHA 2020). Toxic decomposition products such as sulfur oxyfluorides may develop if sulfur hexafluoride is used as an insulating gas under a high thermal impact (for example arcs, a thermal input higher than 500 °C and electrical discharges); the following substances were identified: SF_4_, SF_2_, S_2_F_10_, SO_2_, SOF_2_, SOF_4_, SO_2_F_2_, SOF_10_, S_2_O_2_F_10_, HF and H_2_S (Dervos and Vassiliou 2000). Some of these reaction products such as disulfur decafluoride (S_2_F_10_) cause severe irritation of the eyes, nose and throat and lead to oedema in the lungs and other lung damage (Averyt 2006; Henschler 1972). These toxic reaction products are known to form in certain applications, and guidelines have been adopted to regulate their handling and to ensure occupational safety and health at these workplaces (DGUV 2019).

Recycled sulfur hexafluoride must be handled as specified in the DIN EN 60480 standard for the re-use of sulfur hexafluoride gas (Dilo Armaturen und Anlagen GmbH 2022). Likewise, the limit values for the reaction products should not be exceeded. The limit values for several common impurities that were valid at the time of publication of this addendum are shown in Table 1.

**Tab. 1:** Limit values of some decomposition products or impurities of sulfur hexafluoride

Substance	CAS No.	Type of limit value	Limit value	References
S_2_F_10_	5714-22-7	momentary value	0.01 ml/m^3^	OSHA 2022
SF_4_	7783-60-0	momentary value	0.1 ml/m^3^	OSHA 2021
SO_2_	7446-09-5	8-hour mean	1 ml/m^3^	DFG 2022
HF	7664-39-3	8-hour mean	1 ml/m^3^	DFG 2022
SO_2_F_2_	2699-79-8	8-hour mean	10 mg/m^3^	AGS 2022

The purity of commercial products is at least 98.5%; for use in switchgears, the purity required is 99.995% to 99.999% (Linde GmbH 2008, 2012).

In medicine, sulfur hexafluoride is used as a gas tamponade (about 20%) in eye surgery or as a contrast agent for ultrasound imaging in the form of a microbubble suspension (SonoVue^®^). The contrast agent is made by mixing 0.9% sodium chloride solution with a powder consisting of macrogol 4000, distearoylphosphatidylcholine, sodium dipalmitoylphosphatidylglycerol and palmitic acid in an atmosphere of sulfur hexafluoride. The two components are combined directly prior to use and shaken for 20 seconds; the resulting suspension is injected immediately. Sulfur hexafluoride increases the echogenicity of the blood, which leads to an improved signal-to-noise ratio. The microbubbles formed have a mean diameter of 2.5 µm; 90% have a diameter of less than 6 µm and 99% have a diameter of less than 11 µm and are surrounded by a lipid envelope. The bubbles pass freely into the capillaries because they are about the same size as erythrocytes. After sulfur hexafluoride has entered the body, it is eliminated unchanged with the exhaled air (Bracco 2021; EMA 2006).

A blood:air partition coefficient of 0.05 was calculated using the method of Buist et al. (2012).

The density of gaseous sulfur hexafluoride is about 5 times as high as that of air. Therefore, sulfur hexafluoride may accumulate in low-lying areas of buildings, displacing the atmospheric oxygen and thereby leading to suffocation (DGUV 2019).

Sulfur hexafluoride does not damage the ozone layer but is a very potent greenhouse gas with a greenhouse gas potential (GWP according to EU Regulation No. 517/2014) of 22 800. It is regulated by the Kyoto Protocol. As a result, sulfur hexafluoride emissions should be strictly avoided (DGUV 2019; European Parliament and European Council 2014; Fang et al. 2013).

## 1 Toxic Effects and Mode of Action

Under normal conditions, sulfur hexafluoride is as inert as helium or nitrogen.

In humans, acute exposure to sulfur hexafluoride causes sleepiness at concentrations of 55% (550 000 ml/m^3^) and above and reduces motor and cognitive performance at concentrations of 590 000 ml/m^3^ and above. Inhalation studies with exposure of rats to sulfur hexafluoride for 28 days and 13 weeks did not reveal either local or systemic effects at concentrations of up to 50 215 ml/m^3^. Sulfur hexafluoride was not genotoxic in the in vitro or in vivo studies available. In a developmental toxicity study in rats, sulfur hexafluoride (purity: 99.9999%) slightly increased the incidences of short supernumerary 14^th^ ribs in the foetuses at a concentration of 19 100 ml/m^3^; this was considered a transient delay in development. Teratogenic effects were not observed.

There are no studies available for sensitizing effects on the skin or respiratory tract or for carcinogenicity.

## 2 Mechanism of Action

The (mild) sleepiness observed after acute exposure to sulfur hexafluoride concentrations of 550 000 ml/m^3^ and above can be explained by reduced oxygen uptake. This effect is rapidly reversible after the end of exposure because sulfur hexafluoride is exhaled within a few minutes (ECHA 2020).

A study investigated whether sulfur hexafluoride has cardiac sensitizing potential in dogs after the heart rate was increased by intravenous injection of adrenaline. Two minutes after the recording of the electrocardiogram (ECG) started, 12 dogs were injected with an adrenaline dose of 0.8 µg/kg body weight. After another 5 minutes, the animals were exposed to 20% sulfur hexafluoride via a face mask. Another adrenaline dose of 0.8 µg/kg body weight was injected 5 minutes after the beginning of exposure to sulfur hexafluoride. ECG recording ended after another 5 minutes at the end of the exposure to sulfur hexafluoride; that is, after a total of 17 minutes. Sulfur hexafluoride had no effect on the ECG (ECHA 2020).

The minimum alveolar concentration of sulfur hexafluoride necessary to eliminate movements in response to a painful stimulus (anaesthetic potential) was investigated in 5 dogs (no other details). This value was determined to be 4965 hPa sulfur hexafluoride after exposure for 30 minutes (ECHA 2020).

## 3 Toxicokinetics and Metabolism

### 3.1 Absorption, distribution, elimination

Under normal conditions, sulfur hexafluoride is a gas. It is absorbed via inhalation and exhaled very rapidly in unchanged form (ECHA 2020).

When 12 volunteers (7 men and 5 women) were injected intravenously with stabilized microbubbles of sulfur hexafluoride (SonoVue^®^; 0.03 and 0.3 ml/kg body weight (sulfur hexafluoride doses of 1.2 and 12 µg/kg body weight)), 40% to 50% of the administered dose was exhaled within 1 minute and 78% to 89% was exhaled within 11 minutes irrespective of the dose. No differences between men and women were found and substance-induced effects were not observed (ECHA 2020). SonoVue^®^ is safe for use by humans with impaired renal function because sulfur hexafluoride is rapidly exhaled after the injection of SonoVue^®^ (Bracco 2021).

Rabbits exhaled 80% of stabilized microbubbles within 1 minute after intravenous injection (0.3 and 1.0 ml/kg body weight (sulfur hexafluoride doses of 12 and 40 µg/kg body weight)). After 6 minutes, the amount of sulfur hexafluoride excreted was so high that the level in blood was below the limit of detection of 0.1 ng/ml. The rate of clearance from the blood of rabbits was 218 to 231 ml/min/kg body weight with a half-life of less than 1 minute. Almost all of the sulfur hexafluoride is exhaled in unchanged form; only 0.001% of the administered dose was found in the urine that was collected from the bladder 2 hours after administration of the test substance. Therefore, sulfur hexafluoride does not accumulate in the body (ECHA 2020).

In an inhalation study carried out according to OECD Test Guideline 412, rats were exposed to sulfur hexafluoride in a concentration of 50 215 ml/m^3^ for 6 hours a day for 28 days. About 4 hours after the beginning of the exposure on day 28, the concentration of sulfur hexafluoride was 1.3 to 3.6 µg/ml blood (2.3 ± 0.7 µg/ml blood). Earlier studies detected small amounts of sulfur hexafluoride also in the perirenal adipose tissue of male Wistar rats following inhalation exposure (concentration not reported). The saturation concentration was reached in the perirenal adipose tissue after exposure for 5 hours; 95% of the substance was eliminated within 4 hours. The concentration of sulfur hexafluoride in the exhaled air rapidly decreased (TNO 2009).

Experimental data for the absorption of sulfur hexafluoride through the skin are not available. The IH SkinPerm v2.04 software (AIHA 2019) was used to calculate the amount of test substance that could potentially be absorbed from the gas phase. The model predicted that 4.3 µg would be absorbed through the skin after whole-body exposure (area: 18 000 cm^2^; temperature: 25 °C) at the level of the MAK value of 5000 ml/m^3^ (30 300 mg/m^3^).

### 3.2 Metabolism

Sulfur hexafluoride is not metabolized (ECHA 2020).

## 4 Effects in Humans

### Single exposures

The narcotic influence of sulfur hexafluoride on the mental and psychomotor performance of 9 male volunteers aged between 28 and 37 years and weighing between 70 and 95 kg was investigated in a pressure chamber at normal atmospheric pressure. The test substance was described as of the “highest purity available” (no other details). The test persons were semi-professional divers who were familiar with mouthpiece breathing and mild nitrogen narcosis caused by hyperbaric air. The control tests were performed with air and nitrous oxide. Psychomotor and cognitive abilities and perceptual speed were assessed using a computerized test programme that allowed the persons to perform the tests at their own speed. The results were evaluated on the basis of the response speed and the correctness of the answers. The test battery included tests assessing choice reaction time, colour–word interference reaction time, spatial perception, perceptual speed, working memory performance and the ability to draw logical conclusions. A financial reward was given for the best test results per run to maintain motivation. The test persons were exposed to air and various normoxic gas mixtures: 13%, 26% or 39% nitrous oxide and 39%, 59% or 79% sulfur hexafluoride; one substance was investigated per day and the volunteers completed 5 run-throughs of the computer programme (duration: 12–13 minutes). The first run was carried out with a simulated air control, followed by a control run for the test and 3 test runs with the exposure concentrations in a randomized order, either with nitrous oxide or with sulfur hexafluoride. At the end of the test, the persons were asked to determine the gas concentrations based on their perception. All participants were able to identify the two high nitrous oxide concentrations correctly whereas 2 test persons were not able to differentiate between the low nitrous oxide concentration (13%) and the control. After exposure to sulfur hexafluoride, one participant was not able to differentiate between the control and 39% sulfur hexafluoride. None of the volunteers reported having perceived an odour or a taste, none complained of feeling unwell during exposure. Subjective recovery from the test situation occurred within 2 minutes after the end of the test. Exposure to nitrous oxide affected performance in a concentration-dependent manner and to a statistically significant degree. Depending on the test, about 35% to 68% of the answers were correct after exposure to the high concentration. Exposure of the persons to the three sulfur hexafluoride concentrations reduced test performance by 5% (without statistical significance), 10% or 18% (with statistical significance) (Östlund et al. 1992).

A similar comparative study used a computerized test programme to investigate the psychomotor and cognitive abilities and perceptual speed of 9 male volunteers aged between 30 and 35 years and weighing between 62 and 82 kg in a pressure chamber. The test persons were again semi-professional divers. Three practice rounds were completed, each comprising 3 run-throughs of the computer programme. The programme was made up of 3 tests of varying complexity. This was followed by 1 run-through with the test gas (mixture). As in the earlier study, the test battery included tests assessing choice reaction time, perceptual speed and working memory performance. The response speed and the correctness of the answers were evaluated. A financial reward was given for the best test results per run to maintain motivation. The test persons were exposed to sulfur hexafluoride with a partial pressure of 0, 52, 104 or 156 kPa or to nitrogen with a partial pressure of 103, 575, 825 or 1075 kPa. Data from a previous study (not described in more detail) were included for exposure to nitrous oxide with a partial pressure of 0, 13, 26 or 39 kPa. Each gas was tested on a different day and the concentrations were presented in random order. After exposure to the three sulfur hexafluoride concentrations, the number of correct answers was reduced by 8%, 24% or 49% and the reaction time was delayed (by about 20% at the middle concentration and 70% at the high concentration; estimated from a figure). The effect concentrations for nitrogen, sulfur hexafluoride and nitrous oxide at which performance was decreased by 20% were 830, 97 and 21.5 kPa, respectively. The order of the substances in terms of their narcotic effect is the same as that of their fat solubility (Östlund et al. 1994).

In a storage tower for sulfur hexafluoride, 2 electrical maintenance workers were exposed to sulfur hexafluoride and by-products. The two workers fell unconscious while performing maintenance work in the storage tower and were exposed for 19 and 30 minutes, respectively. After both workers had been rescued, the oxygen concentration was found to be 21% at the top of the tower and 18.6% at the bottom. Without specifying the sampling site, the sulfur hexafluoride concentration was reported to have been 1500 ml/m^3^ and that of SO_2_F_2_ 50 ml/m^3^. Tests for other fluorides or oxyfluorides were not carried out due to time constraints. The worker who had been exposed for 30 minutes developed frank pulmonary oedema 90 minutes after the end of exposure while he was being transported to hospital. The pulmonary oedema was probably caused by toxic by-products that were formed by sparks or electrical arcs and required oxygen therapy. This worker had mild peripheral cyanosis and was coughing up blood at that time. The breathing problems had subsided by the third day after the accident. During the 2 weeks that followed, he had difficulty concentrating, an altered sleep pattern and was lethargic. He was symptom-free 6 months after the accident. The worker who had been exposed for a shorter period was also treated with oxygen in the ambulance, but had no breathing problems or cyanosis (Pilling and Jones 1988).

A study compared sulfur hexafluoride and nitrous oxide in terms of their potential for inducing anaesthesia by determining the pain threshold, subjective sensations and the length of recovery. In this test, 20 young adults were exposed to concentrations of up to 79% sulfur hexafluoride and 21% oxygen or 30% nitrous oxide (no other details) for 10 minutes. The pain threshold was increased by an average of 19% after exposure to sulfur hexafluoride and in the same persons by 22% after exposure to nitrous oxide. The earliest subjective sensation appeared when sulfur hexafluoride reached a concentration of 55%. Sleepiness, analgesia and a deepening of the voice were reported. The deepening of the voice was probably due to the higher density of sulfur hexafluoride in comparison with air. The persons who had been exposed to sulfur hexafluoride recovered faster from the symptoms than those who had been exposed to nitrous oxide. Four persons reported shortness of breath (ECHA 2020).

In 58 volunteers who were exposed to 70% sulfur hexafluoride and 30% oxygen, no effects were observed apart from a transient deepening of the voice. The study investigated the differences in the respiratory flow rate between smokers (n = 29) and non-smokers (n = 29) (ECHA 2020).

Two authors tested themselves by breathing in a mixture of 78% sulfur hexafluoride and 22% oxygen for a short period of time (no other details). They reported experiencing a cool sensation in the upper respiratory tract and a deepening of their voices. After taking 20 to 30 breaths at a normal rate and volume, they experienced mild vertigo that disappeared after a few minutes of breathing normal air (ECHA 2020).

In a study that investigated the effects of carrier gas on the deposition of triphenyl phosphate particles in the lungs, 2 male and 2 female volunteers were exposed to 1 litre of a mixture of 80% sulfur hexafluoride and 20% oxygen; it was not reported how long exposure lasted. No adverse effects by sulfur hexafluoride were reported (ECHA 2020).

### Allergenic effects

#### Sensitizing effects on the skin

There are no data available.

#### Sensitizing effects on the airways

There are no studies available for sensitizing effects on the airways. The reports described above did not provide evidence of sensitizing effects of sulfur hexafluoride on the airways.

#### Other effects

Several reports describe anaphylactoid reactions after intravenous injection of an ultrasound contrast medium containing sulfur hexafluoride and macrogol 4000 (see Introduction for composition).

A 59-year-old male patient had an anaphylactic reaction and other symptoms. The patient was atopic, but had no previous history of drug allergies. The contrast agent was administered for an ultrasound scan carried out as a follow-up for a percutaneous coronary intervention. The patient showed signs of an anaphylactic reaction a few minutes after infusion. He had probably been exposed to the same contrast agent 2 months before when an earlier ultrasound scan was taken. Extensive tests were carried out to determine the cause of the reaction. In the prick test, the contrast agent, macrogol 4000, macrogol 3350 and macrogol 400 (pure substance in each case) yielded positive results. In a basophil activation test that analysed the expression of CD63 as a basophil activation marker, negative results were obtained with macrogol 4000 in concentrations of 1 and 0.5 mg/ml. The results of a prick test with Tween 80 (0.04 mg/ml) and of an intradermal test with Tween 80 (0.004 mg/ml) yielded negative results. A prick test with macrogol 4000 produced negative results in 10 control persons. Therefore, the authors assumed that macrogol 4000 caused the anaphylactic reaction (Oyarzabal et al. 2017).

A 62-year-old patient complained of shortness of breath, profuse sweating and a sensation of intense heat that developed within about 30 seconds after the injection of 1.5 ml SonoVue^®^. The patient fell unconscious a few minutes later. These symptoms had not occurred after the first injection 3 months before. No reactions to the contrast agent or macrogol 4000 were observed in a subsequent prick test. It was not possible to identify what caused the symptoms (Coudray et al. 2017).

In a similar case, a 39-year-old female patient likewise developed signs of a severe anaphylactic reaction immediately after administration of the contrast agent. Three months later, prick tests and intradermal tests were carried out with increasing concentrations of the contrast agent to avoid a severe allergic reaction during testing. However, all skin tests yielded negative results. Provocation was not included in the tests to avoid the risk of another anaphylactic reaction. As it was the first contact with the contrast agent, the authors concluded from the symptoms of the reaction and the results of the prick and intradermal tests that the reaction had not been mediated by IgE. However, the mechanism remains unclear (Levano et al. 2012).

Symptoms of an allergic reaction were observed in a 60-year-old male patient following another ultrasound scan using the above-mentioned contrast agent. A Kounis syndrome was assumed, but as in the case described above, the constituent that caused the reaction was not investigated (van Ginkel et al. 2014).

Likewise, a 45-year-old female patient without pre-existing conditions had a severe reaction to a contrast agent containing sulfur hexafluoride. She developed symptoms such as urticaria with oedema on her face, hands and limbs, which persisted up to the following day. No subsequent tests were carried out to identify the constituent that caused the reaction (Solivetti et al. 2012).

Three other cases of patients were reported in the Netherlands. The patients, who had underlying cardiac conditions, developed severe anaphylactoid reactions to a contrast agent containing sulfur hexafluoride. The first case involved a 59-year-old female patient who produced a reaction to the contrast agent in epicutaneous and intracutaneous allergy tests; no reaction was observed in 5 control persons. The authors suggest that this may have been a non-specific reaction to the macrogol 4000 excipient. However, no other tests were carried out. In the second case of a 70-year-old female patient, no allergy tests were carried out. Dermatological tests carried out in a third, 80-year-old male patient found no evidence of an allergic reaction. In this case, too, a non-specific anaphylactoid reaction was assumed (de Groot et al. 2004).

A study investigated adverse effects after contrast-enhanced ultrasonography using contrast agents containing sulfur hexafluoride. Adverse effects were observed in 7 of 352 patients (2.0%) with heart disease who were administered the contrast agent within a period of 4 years. Four patients (1.1%) developed mild allergic reactions that were manifest in the form of skin erythema and mild sinus tachycardia. Three patients (0.9%) developed severe allergic reactions and suffered a (non-fatal) shock. No other tests were carried out. The authors suggest that sulfur hexafluoride and macrogol 4000 may have caused the allergic reactions (Geleijnse et al. 2009). However, the publication described by the authors is not suitable for assessing the effects of the contrast agent containing sulfur hexafluoride because it was for sulfur dioxide and hydrogen sulfide.

Cases of allergic or hypersensitivity reactions that were explicitly caused by sulfur hexafluoride have not been described in the literature. The systemic reactions that were observed after administration of the contrast agent containing sulfur hexafluoride seem to have been caused by the macrogol 4000 excipient. Many cases of hypersensitivity and anaphylactic reactions to macrogols have been described in different contexts (Wenande and Garvey 2016).

## 5 Animal Experiments and in vitro Studies

### 5.1 Acute toxicity

#### 5.1.1 Inhalation

The studies of acute inhalation toxicity are shown in Table 2. These are earlier studies that do not meet current requirements. However, no substance-induced findings were observed in the inhalation studies carried out in 2009 and 2016 according to OECD test guidelines with repeated exposure of rats to sulfur hexafluoride concentrations of up to 20 052 and 50 215 ml/m^3^, respectively (see Section 5.2.1). Therefore, the substance does not cause acute toxicity up to these concentrations.

**Tab. 2:** Acute inhalation exposure to sulfur hexafluoride (study inadequately described; ECHA 2020)

Species number, strain	Exposure	Toxicity	Comments
rat, no other details	17% SF_6_, 18 hours (n = 6)	no unusual findings, no mortality	
	80% SF_6_, 2 hours, 23 hours (n = 5)	no unusual findings, no mortality	
	80% SF_6_ + 20% O_2_, 16–24 hours (n = 50)	no unusual findings, no mortality	
rat, no other details	1 500 000 mg/m^3^ (25%) SF_6_	no mortality, neurobehavioural abnormalities, slower reflexes, passive avoidance acquired more slowly	effects caused by low level of O_2_ in the atmosphere (15%)
	300 000 mg/m^3^ (5%) SF_6_	no mortality, impairment of short-term and long-term memory	
	62 900 mg/m^3^ (1%) SF_6_	no unusual findings, no mortality	
rat, 17 (no other details)	72% SF_6_ / 28% O_2_ (v/v), 6 hours	no unusual findings, no mortality	
	a second exposure to 78% SF_6_ / 22% O_2_ (v/v) 40 days after the first exposure, 6 hours	no unusual findings, no mortality	

SF_6_: sulfur hexafluoride; O_2_: oxygen

#### 5.1.2 Oral administration

There are no studies available.

#### 5.1.3 Dermal application

There are no studies available.

### 5.2 Subacute, subchronic and chronic toxicity

#### 5.2.1 Inhalation

A 28-day inhalation study carried out according to OECD Test Guideline 412 included a screening test for toxic effects on reproduction according to OECD Test Guideline 422. Groups of 12 male and 12 female Wistar rats were exposed nose-only to sulfur hexafluoride concentrations of 0 or 50 215 ml/m^3^ (302 687 mg/m^3^; purity: 99.999%) daily for 28 days. However, the animals were in fact whole-body exposed because of leakage. The animals were exposed for 6 hours a day, the males from at least 2 weeks prior to mating and during mating, overall for at least 28 days, and the females from at least 2 weeks prior to mating and during gestation up to day 19. The offspring were born naturally and examined together with the dams on postnatal day 4; the dams were additionally evaluated histopathologically. Functional observational battery (FOB) tests were carried out with 5 animals per sex and group. The tests were performed on the males shortly before the end of the exposure period and on the females on day 4 of lactation. The tests included 24 parameters from 7 functional groups. Statistically significant differences between the exposed animals and control animals were obtained for two individual parameters. The landing foot splay (distance between the 4^th^ toes of the hind paws when landing from a height of about 30 cm) was reduced in the exposed animals. However, these values were far below the historical control values in both the control and exposed animals. Therefore, this finding is considered incidental. The motor activity levels of the male control animals were markedly below the historical control values, whereas the motor activity levels of the exposed animals were in the range of those of the historical controls. Therefore, this difference between the exposed and non-exposed animals is also regarded as an incidental finding. No other substance-induced findings were observed in the parental animals. Therefore, a NOAEC (no observed adverse effect concentration) of 50 215 ml/m^3^ was proposed for the effects of sulfur hexafluoride on the parental animals (TNO 2009).

The landing foot splay is only one of many parameters that can be used to study neuromuscular function. If the substance has neurological effects, exposed animals are more likely to exhibit reduced motor activity. Likewise, other parameters tested by the FOB would have been affected. Therefore, the Commission agrees that sulfur hexafluoride does not induce effects up to a concentration of 50 215 ml/m^3^.

In an inhalation study carried out according to OECD Test Guideline 413 that was published in 2016, groups of 10 male and 10 female Wistar rats were exposed nose-only to sulfur hexafluoride concentrations of 0 or 20 052 ml/m^3^ (purity: 99.999%) for 6 hours a day, on 5 days a week, for 13 weeks. The body weight gains of the females were slightly reduced on days 20, 52 and 92. The decreases were statistically significant only in a few cases. No other substance-induced findings were observed. Therefore, a NOAEC of 20 052 ml/m^3^ was derived from the findings of this study (Triskelion 2016).

A 4-month inhalation study was carried out in the Soviet Union in 1981 with rats and guinea pigs. Effects on the kidneys and the central nervous system were described at sulfur hexafluoride concentrations of 9800 mg/m^3^ (1619 ml/m^3^) and 77 000 mg/m^3^ (12 721 ml/m^3^). Many details of the study, such as the purity of the substance and the number and sex of the examined animals, were not reported and the animals were not examined histopathologically (ECHA 2020). This study has not been included in the evaluation because the results are not consistent with recent, well-documented studies that were carried out according to test guidelines.

#### 5.2.2 Oral administration

There are no studies available.

#### 5.2.3 Dermal application

There are no studies available.

### 5.3 Local effects on skin and mucous membranes

#### 5.3.1 Skin

There are no studies available.

#### 5.3.2 Eyes

There are no studies available that investigated irritation of the eyes.

Ophthalmological examinations that were performed as part of acute inhalation studies and a 13-week study did not detect any effects (see Section 5.1.1 and 5.2.1) (ECHA 2020; Triskelion 2016).

### 5.4 Allergenic effects

#### 5.4.1 Sensitizing effects on the skin

There are no specific studies available for sulfur hexafluoride, which is gaseous at room temperature.

#### 5.4.2 Sensitizing effects on the airways

There are no studies available.

### 5.5 Reproductive and developmental toxicity

#### 5.5.1 Fertility

The 28-day inhalation study carried out in 2009 according to OECD Test Guideline 422 in Wistar rats that was described in Section 5.2.1 did not reveal any substance-induced changes in the reproductive organs, sperm parameters, mating parameters or in the number of corpora lutea or implantations following daily exposure to a sulfur hexafluoride concentration of 50 215 ml/m^3^ (purity: 99.999%) (ECHA 2020; TNO 2009).

#### 5.5.2 Developmental toxicity

In a developmental toxicity study carried out according to OECD Test Guideline 414, 22 pregnant RccHan^®^WIST rats per concentration group were exposed nose-only to analysed concentrations of 0 (air), 10 100 or 19 100 ml/m^3^ for 6 hours daily from days 6 to 19 of gestation. The test concentrations were chosen on the basis of the study carried out according to OECD Test Guideline 422 and the 13-week inhalation study. These studies were carried out as limit tests with 50 215 and 20 052 ml/m^3^, respectively. As no substance-induced effects were observed in either study, concentrations of 10 000 and 20 000 ml/m^3^ were selected as target concentrations for the developmental toxicity study. The higher concentration was based on the limit for classification (categories 2 and 3) of 20 000 ml/m^3^ established by the United Nations Globally Harmonized System of Classification and Labelling of Chemicals for specific acute target organ toxicity of gaseous substances (UNECE 2021). Sulfur hexafluoride with a purity of 99.9999% was used. The dams underwent caesarean section on day 20 of gestation. The dams did not exhibit any unusual behaviour. The body weight gains, gravid uterine weights, adjusted body weights of the dams, feed consumption, and the gross-pathological examination of the organs did not reveal any substance-induced effects. The weights of the thyroid and parathyroid glands, the histopathological examination of these glands and the T3, T4 and TSH levels in the blood were in the normal range. Likewise, there were no substance-induced effects on the number of implantations or resorptions, the sex ratio, litter size, embryofoetal development as assessed by anogenital distance, placental, litter and foetal weights or foetal abnormalities. The incidences of short supernumerary 14^th^ ribs were slightly increased in the foetuses of the high concentration group. This finding was not considered adverse because the increase was slight and the delay in growth only temporary and thus did not result in a malformation. Therefore, the sulfur hexafluoride concentration of 19 100 ml/m^3^ is regarded as the NOAEC for developmental toxicity (Labcorp Early Development Laboratories Ltd 2021). The concentration of 19 100 ml/m^3^ is also the NOAEC for maternal toxicity.

The 28-day inhalation study (OECD Test Guideline 412) carried out in 2009 with Wistar rats that included a screening test for toxic effects on reproduction according to OECD Test Guideline 422 (described above in Section 5.2.1) did not detect any substance-induced changes in the dams or offspring following daily exposure to a sulfur hexafluoride concentration of 50 215 ml/m^3^ (purity: 99.999%) (TNO 2009). An external examination of the offspring was performed in the screening test carried out according to OECD Test Guideline 422, but no skeletal or visceral examinations. Therefore, this study does not include a full evaluation of teratogenicity.

A toxicity study with repeated exposure was combined with a screening test for reproductive and developmental toxicity that complied with the ICH Test Guideline for the testing of medicinal products to investigate the effects induced by the ultrasound contrast agent SonoVue^®^ (see Introduction for composition) after administration to Sprague Dawley rats by intravenous bolus injection (ECHA 2020). The substance was administered to groups of 22 male rats daily for 4 weeks before and during mating for a total period of 7 weeks and to groups of 22 female rats for 4 weeks before and during mating and up to day 17 of gestation. The contrast agent contained 6.4 µl of stabilized sulfur hexafluoride microbubbles per ml of physiological saline (solid–liquid suspension) and was injected into the tail vein. The administered doses were 0, 0.2, 1 or 5 ml/kg body weight and day (microbubble doses of 0, 1.28, 6.4, 32 µl/kg body weight and day). Due to the large amount of substance being administered, the high dose was given by pump at a rate of 2 ml/minute; the other doses were administered by hand within a period of about 75 seconds. The female rats underwent necropsy on day 20 of assumed gestation, and the foetuses were examined for visceral and skeletal anomalies. No substance-induced effects were observed in the parental animals (systemic effects or effects on reproduction) or in the offspring (embryotoxicity, foetotoxicity or teratogenicity), and there were no effects on fertility, reproductive behaviour, the oestrous cycle or on reproductive parameters. In this study, the NOAEL (no observed adverse effect level) was 5 ml/kg body weight and day. If 1 ml suspension contains 45 µg sulfur hexafluoride, as reported in EMA (2006), this corresponds to a dose of 225 µg/kg body weight and day (ECHA 2020).

### 5.6 Genotoxicity

#### 5.6.1 In vitro

Sulfur hexafluoride did not induce mutations in the Salmonella typhimurium strains TA98, TA100, TA1535 and TA1537 or in Escherichia coli WP2uvrA either in the presence or in the absence of a metabolic activation system. This study from 2010 was carried out according to OECD Test Guideline 471 in incubator chambers with 19% oxygen, 5% carbon dioxide and up to 76% sulfur hexafluoride (diluted in nitrogen). Positive and negative controls verified the functioning of the test system, and the analysis of the exposure concentration at the end of the 24-hour incubation period confirmed that the test substance had the desired concentration. Sulfur hexafluoride did not induce cytotoxicity (ECHA 2020).

Likewise, a TK^+/–^ test in L5178Y mouse lymphoma cells carried out in 2010 according to OECD Test Guideline 476 yielded negative results in the absence of a metabolic activation system after 4 or 24 hours as well as in the presence of a metabolic activation system after 4-hour incubation with the test substance. This study, too, was carried out in incubator chambers with 19% oxygen, 5% carbon dioxide and up to 76% sulfur hexafluoride (diluted in nitrogen). Positive and negative controls verified the functioning of the test system. The exposure concentration was analysed following incubation for 24 hours and confirmed that the test substance had the desired concentration. Sulfur hexafluoride did not induce cytotoxicity (ECHA 2020).

#### 5.6.2 In vivo

The 28-day inhalation study described in Section 5.2.1 included a micronucleus test in the reticulocytes of male Wistar rats that was carried out according to OECD Test Guideline 474. This test yielded negative results after exposure to sulfur hexafluoride concentrations of 0 or 50 215 ml/m^3^ (purity: 99.999%). Five male rats from the control and sulfur hexafluoride groups were examined as were 5 animals from a positive control group that were given single intraperitoneal injections of mitomycin C 24 hours prior to analysis. The exposed animals were examined for micronuclei within 24 hours after the last exposure, and the ratio of polychromatic to normochromatic erythrocytes was determined. Cytotoxicity did not occur in the study. The sulfur hexafluoride concentration in the blood of the exposed animals was between 1.3 and 3.6 µg/ml (mean: 2.3 ± 0.7 µg/ml). Therefore, it is assumed that the substance reached the bone marrow (TNO 2009).

An inadequately described study found no evidence of micronuclei after the exposure of rats to a single sulfur hexafluoride concentration of 250 000 ml/m^3^ or repeated exposure to a concentration of 12 800 ml/m^3^ (no other details) (ECHA 2020).

### 5.7 Carcinogenicity

There are no data available.

## 6 Manifesto (MAK value/classification)

Sulfur hexafluoride (purity: 99.999%) did not induce effects in rats up to a concentration of 50 215 ml/m^3^. Acute pre-narcotic effects were observed in volunteers after exposure to concentrations 10 times as high. Toxic reaction products are known to form during certain applications; guidelines have been established that regulate the handling of the substance and ensure occupational safety and health at these workplaces (DGUV 2019).

**MAK value. **The acute narcotic potency of sulfur hexafluoride was compared with that of nitrous oxide in two controlled volunteer studies (Östlund et al. 1992, 1994). Both studies suggest that the acute effects of nitrous oxide on motor and cognitive performance are 4 to 5 times as strong as those induced by sulfur hexafluoride. In isolated cases, performance was significantly reduced after exposure to 39% sulfur hexafluoride / 21% oxygen (sulfur hexafluoride concentration of 390 000 ml/m^3^); however, after considering all studies together, performance was not reduced with statistical significance at a concentration of 390 000 ml/m^3^, but only at concentrations of 590 000 ml/m^3^ and above.

Sulfur hexafluoride with a purity of 99.999% did not induce effects or irritation in rats after exposure by inhalation for 13 weeks at a concentration of 20 052 ml/m^3^ (5 days a week, nose-only) (Triskelion 2016) or after whole-body exposure to 50 215 ml/m^3^ for 28 days (TNO 2009). A MAK value of 5000 ml/m^3^ is calculated from the NOAEC of 20 052 ml/m^3^ after extrapolating the data from animal studies to humans (1:2) and taking into consideration a possible intensification of the effects over time (1:2). This MAK value is valid for pure sulfur hexafluoride. A blood:air partition coefficient of 0.05 was calculated using the formula of Buist et al. (2012). Therefore, the increased respiratory volume at the workplace does not have to be considered for the derivation of the MAK value.

**Peak limitation. **Sulfur hexafluoride remains classified in Peak Limitation Category II because pure sulfur hexafluoride did not cause irritation in studies with volunteers or in inhalation studies in rats. Likewise, the excursion factor of 8 has been retained because performance was reduced in humans in isolated cases only at concentrations of 390 000 ml/m^3^ and above. However, overall, the findings were not statistically significant.

**Prenatal toxicity. **A developmental toxicity study in rats carried out according to OECD Test Guideline 414 did not reveal substance-induced findings in the dams up to the high sulfur hexafluoride (purity: 99.9999%) concentration of 19 100 ml/m^3^. The slight increase in the incidences of short supernumerary 14^th^ ribs in the foetuses is not regarded as adverse (Labcorp Early Development Laboratories Ltd 2021) because it was a transient delay in development (DeSesso and Scialli 2018; Solecki et al. 2013). Therefore, the NOAEC for developmental and maternal toxicity is 19 100 ml/m^3^. A study carried out according to OECD Test Guideline 422 did not detect any effects on the dams or offspring even at a concentration of 50 215 ml/m^3^ (TNO 2009).

Teratogenicity was not observed. The highest concentration of 19 100 ml/m^3^ was at the limit for classification (categories 2 and 3) of 20 000 ml/m^3^ established by the United Nations Globally Harmonized System of Classification and Labelling of Chemicals for specific acute target organ toxicity of gaseous substances (UNECE 2021). Likewise, the screening study carried out according to OECD Test Guideline 422 did not detect perinatal toxicity at a sulfur hexafluoride concentration of 50 215 ml/m^3^. On this basis, the margin between the NOAEC of 19 100 ml/m^3^ and the MAK value of 5000 ml/m^3^ of a factor of 4 is regarded as sufficiently large. The increased respiratory volume does not have to be taken into account (see above). As a result, sulfur hexafluoride has been classified in Pregnancy Risk Group C.

**Carcinogenicity. **Carcinogenicity studies are not available. Carcinogenic effects are unlikely to occur because sulfur hexafluoride is neither genotoxic nor reactive and is rapidly eliminated. Therefore, sulfur hexafluoride has not been classified in any of the categories for carcinogens.

**Germ cell mutagenicity. **There are no studies in germ cells. The studies that are available found no evidence of genotoxic effects. Therefore, sulfur hexafluoride has not been classified in any of the categories for germ cell mutagens.

**Absorption through the skin. **To date, the findings of animal and volunteer studies that investigated inhalation exposure to the substance do not suggest that sulfur hexafluoride induces systemic toxicity. There are no experimental data available for absorption through the skin. The amount absorbed after exposure at the level of the MAK value (5000 ml/m^3^ ≙ 30 350 mg/m^3^, respiratory volume 10 m^3^) is calculated to be in the gram range even assuming low levels of absorption by inhalation. A model used to calculate the dermal absorption of the substance from the gas phase under these conditions (MAK value, whole-body exposure for 8 hours) found that an amount of 4 µg would additionally be absorbed. This amount is negligible compared with that absorbed by inhalation. Therefore, sulfur hexafluoride has not been designated with an “H” (for substances which can be absorbed through the skin in toxicologically relevant amounts).

**Sensitization. **There are no positive clinical findings or experimental studies available for sensitizing effects on the skin or respiratory tract. As a result, sulfur hexafluoride has not been designated with either “Sh” or “Sa” (for substances which cause sensitization of the skin or airways).
